# Honey as an Alternative Dressing in Post-Palatoplasty—Two Case Reports

**DOI:** 10.1155/2024/8671377

**Published:** 2024-10-26

**Authors:** Clara David, Irfan Rasul, Yossy Y. Ariestiana, Abul Fauzi

**Affiliations:** Department of Oral and Maxillofacial Surgery, Faculty of Dentistry, Universitas Hasanuddin, Makassar 90245, Indonesia

**Keywords:** alternative dressings, honey, palatoplasty, wound healing

## Abstract

Palatoplasty is a surgical procedure used to repair a cleft palate. Postsurgery there are times when the healing process is disrupted. Honey has been utilised since ancient times as an antibacterial, anti-inflammatory, and regenerative treatment for wounds, and it has been shown to expedite the wound healing process by promoting the formation of new tissue.

## 1. Introduction

The palate is divided into three sections: the anterior and posterior hard palatal mucosa protecting bone and the soft palatal mucosa covering muscle in the pharynx [1]. Palatoplasty is a surgical procedure used to repair a cleft palate [2, 3]. Palatoplasty is performed after cleft reconstruction as a second stage of surgery in cleft situations. It is performed in the second year of life when the child is about one and a half years old [4]. The natural healing process following surgery is occasionally interrupted, resulting in persistent wounds and tissue necrosis [5]. Human oral wounds, in general, are asymmetrical in height, width, depth, and tissue ratios because they occur as a result of trauma, cancer surgery, or inadequate postsurgical healing. The heterogeneous anatomy of the palatal mucosa can cause wounds to differ in laterality, completeness, severity, and tissue architecture. Laboratory blood analyses can help assess the quality of healing that follows and determine whether recovery was successful [1]. In this regard, natural components such as honey accelerate the wound healing process by creating new tissue [5].

Honey has been utilized as an antibacterial, anti-inflammatory, and regenerative treatment for wounds since ancient times [6]. Honey is effective against a variety of oral ulcers with multiple pathologies, particularly in the treatment of oral mucositis, and this effect can be attributed to the type of honey used rather than the type of oral health condition, demonstrating that topical application of honey is relatively safe for use across a range of oral health conditions [7]. Numerous preclinical investigations have effectively assessed different honey compositions, including topical application, hydrogel based on honey, and honey dressings based on gauze [6]. Honey's antibacterial properties and high viscosity make it a good barrier against the environment and provide excellent hydration when used topically as a wound dressing solution [8]. Another study has reviewed that honey can accelerate the healing of ulcers in the palate, is efficacious in various chronic wounds, and its use in a low-cost, easily accessible hospital setting with no long-term negative effects would make honey an ideal dressing alternative for patients with palatal wounds [9]. The following explains the use of honey ingredients for palatal wounds and/or palatoplasty compared to other ingredients with their respective outcomes described in Table 1.

## 2. Case Presentation

### 2.1. Case 1

**Figure 1:**
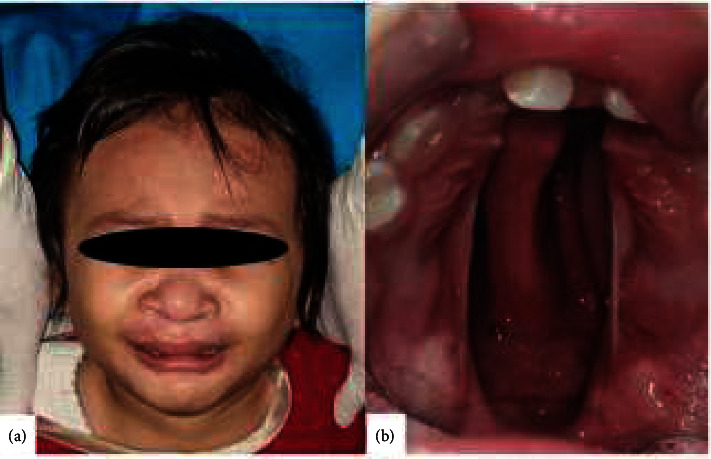
Case 1. (a) Extraoral view. (b) Intraoral view.

A 1.5-year-old female patient was referred to the oral and maxillofacial surgery department with chief complaints of cleft palate present since birth. When consuming food and fluids, even with a spoon-tipped pacifier, she experienced discharge from the nose. The patient had no family history of a similar disorder and no history of milk and food allergies. A primary lip surgery has been done in the three months old. An extraoral examination showed a post-labiaplasty scar on the upper lip, while an intraoral examination revealed a cleft palate that extended from the hard palate to the uvula (Figure 1).

### 2.2. Case 2

**Figure 2:**
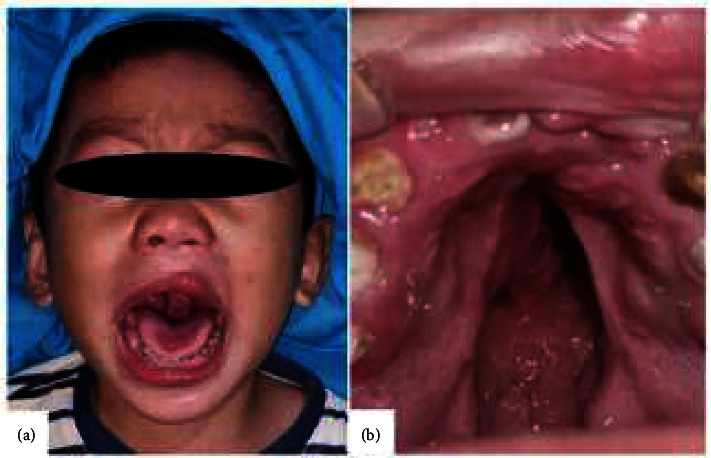
Case 2. (a) Extraoral view. (b) Intraoral view.

A 3.5-year-old male patient was referred to the oral and maxillofacial surgery department with chief complaints of cleft palate present since birth and unclear speech. The patient also experienced discharge from the nose during eating and drinking. A family member, a grandmother, had a similar condition. The patient had no history of milk and food allergies. A primary lip surgery has been done in the four months old. An extraoral examination showed a post-labiaplasty scar on the upper lip, while an intraoral examination revealed a cleft palate that extended from the hard palate to the uvula (Figure 2).

### 2.3. Case Management/Treatment

**Figure 3:**
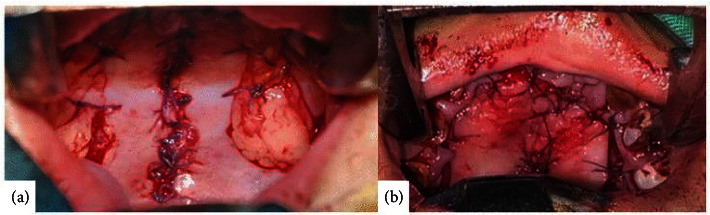
Result palatoplasty. (a) Case 1: palatoplasty with buccal fat pad. (b) Case 2: palatoplasty without buccal fat pad.

Two cleft palate cases are presented in this case report, along with their surgical management and the use of honey as an alternative dressing. Informed consent was obtained from the parents to perform palatoplasty with honey-based dressing. Palatoplasty with two push-back flap technique surgery (Case 1 with buccal fat pad; Case 2 without buccal fat pad) was performed in two cases under general anesthesia (Figure 3). Gabapentin 50 mg was administered 2 h prior to surgery, and ceftriaxone 500 mg an hour prior to surgery. After palatoplasty, honey was applied to sterile gauze on the obturator surgery and suture was performed. The honey used in this case is the result of meliponiculture of the *Trigona* sp. species of stingless bee with the most significant composition of coconut pollen.

**Figure 4:**
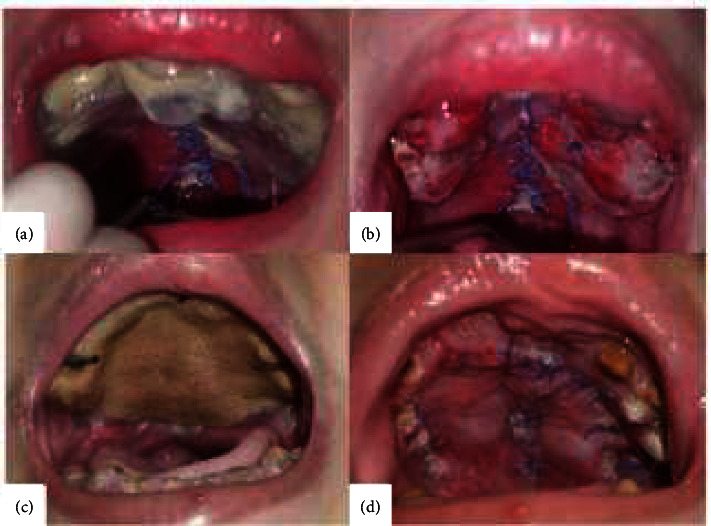
Clinical signs five days; (a) and (c) before removing the obturator; (b) and (d) after removing the obturator with honey as dressing.

**Figure 5:**
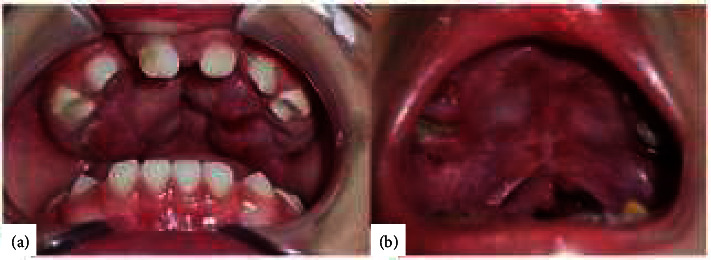
Clinical signs 30 days after palatoplasty with honey as dressing. (a) Case 1. (b) Case 2.

On the fourth day after surgery, an obturator removal control was done at the oral and maxillofacial surgery (Figure 4). The intraoral condition revealed no blood clots and active bleeding with minimum hyperemia. The intraoral condition one month after surgery showed uneventful healing in both cases (Figure 5).

The honey used in this case results from meliponiculture of the *Trigona* sp., a species of stingless bee with the most significant composition of coconut pollen [15]. This honey is one of the natural bioactive ingredients being researched and developed at Hasanuddin University. This honey has gone through the inspection process at the BPOM (Food and Drug Regulatory Agency) and is the standard for food and drug control in Indonesia (appendix 1).

## 3. Discussion

In this case report, the authors share their experience in treating a patient after palatoplasty surgery. The patient was successfully managed using a comprehensive care approach involving the use of edible honey-based dressings and dietary instructions according to the postoperative time, as well as appropriate wound care.

The palatal shelves fail to close and fuse between weeks 8 and 12 of embryonic development, resulting in a cleft of the hard and/or soft palate [14, 15]. The diagnosis of nonsyndromic orofacial cleft and socioeconomic status were significantly correlated in several kinds of published research [15]. In the developed nations, the majority of scientists think that a combination of environmental (e.g., medications, malnourishment, and maternal sickness) and hereditary factors causes clefts [14]. As is the case with these two cases under discussion, the three primary kinds of clefts are isolated cleft palate, cleft lip only, and cleft lip and palate [16]. In addition to repairing the palate's architecture, the primary objective of palatoplasty is to support healthy velopharyngeal function, which in turn creates the necessary conditions for speech production to occur naturally [17].

The main goal of mending is primary union, which is the process by which the tissues approached by surgical sutures or tapes recover with the least amount of tissue loss. These types of wounds heal to leave a thin, tidy scar. Neutrophils start to show up at the incisional edges and move toward the fibrin clot within 24 h. In 24–48 h, the epidermal continuity is restored. By day three, macrophages have mostly taken the place of the neutrophils. Day 5: Maximum neovascularization and granulation tissue fill the incisional space. This underlies the release of obturator surgery on the fifth day after palatoplasty. The second week is characterized by fibroblasts continuing to proliferate and accumulate. By the conclusion of the first month, the scar is composed of an intact epidermis covering cellular connective tissue free of inflammatory infiltrate [18, 19].

Natural proteins in the body called growth factors regulate several important cellular processes involved in the process of proper tissue repair [18]. When tissue damage produces signaling between distinct cell types and skin compartments, physiological wound healing begins. Growth factors and cytokines such as IL 1, and IL 6, tumor necrosis factor (TNF), platelet growth factor (PDGF), and fibroblast growth factor 2 (FGF 2) will be secreted by inflammatory cells. Keratinocytes can migrate between the fibrin clot and the dermis via upregulating integrin and cytoskeletal components and proteases such as matrix metalloproteinases (MMPs). This is known as re-epithelialization, followed by keratinocyte proliferation at the wound border, restoring epidermal stratification. Following that, neoepithelium and granulation tissue migrate to the wound site. Keratinocytes become stimulated and secrete EGF and FGF. More macrophages, fibroblasts, endothelial cells, and macrophages migrate to the wound bed [20].

Palatoplasty techniques with and without a buccal fat pad were used in this study. Pedicled buccal fat pads have been proven in multiple trials to produce positive outcomes, including early epithelialization three to four weeks post-surgery by adding a vascular layer to aid in healing. Because of its abundant vascularity, proximity to the treatment location, little donor-site morbidity, and relatively straightforward surgical technique for covering adjacent abnormalities [21]. Aside from using the buccal fat pad technique, using honey as a dressing substitute for post-palatoplasty patients also accelerates wound healing in the area of the procedure. Honey-based dressing accelerates wound healing time in both patients. Several studies have confirmed the shorter wound healing time when using honey as dressing due to its capability in growth factor stimulation, increased oxygen release from hemoglobin, and stimulation of the activity of macrophages and fibroblasts [5, 9]. Other advantages of topical honey-based dressing are its high viscosity properties, which maintain a moist tissue environment that will benefit the removal of the obturator.

Honey is a complex resource that comes in various forms depending on geography, bee species, and production methods [8]. *Tetragonula biroi*, often known as *Trigona* bees, are stingless bees that live differently from other honey bee species in that they do not only rely on pollen from flowers. *Trigona* bees are bred reasonably easily and anywhere thanks to their distinctive mode of life [22]. Numerous health benefits of stingless bee honey include its ability to heal wounds and its anti-inflammatory, antimicrobial, antibacterial, antidiabetic, and anticancer qualities. It has been suggested that the antioxidative and anti-inflammatory properties of flavonoids and phenolic compounds contribute to the possible positive benefits [23].

Honey will produce H_2_O_2_ and other chemical compounds, such as MGO and Def-1, H_2_O_2_ with antibacterial and regenerative capabilities that may aid in antibiotic resistance and tissue regeneration. Honey's natural antibacterial qualities (H_2_O_2_, osmotic action, and polyphenols) and capacity to insulate the wound site from outside air contribute to its potential to prevent wound infection and hasten wound healing. Elimination in the wound region can encourage tissue regeneration and provide properties that aid healing [8]. This is reinforced by numerous research studies that have examined the health benefits of polyphenols, including their antioxidant and neuroprotective effects on various disorders related to the nervous system [23].

In a series of studies, Kreshanti demonstrated that intraoral honey treatment at the site as oral drops significantly accelerated the lateral palatal defects' epithelialization after palatoplasty and appeared to produce favorable results for the SNA angle in nearly half of the subjects during the early stages of skeletal growth [16, 24]. To analyze every surgical procedure now accessible, we recommend that future studies include a more significant number of patients with a wider range of situations. This study only assessed the wound healing process by clinical appearance; additional research is required to examine the molecular and biochemical pathways involved in wound healing following palatoplasty with honey-based dressing.

The application of an MGH-infused dressing initiated the healing process, improved debridement, and accelerated the cleansing phase of the wound bed. MGH reduced odor and exudate production while maintaining an optimal moist wound bed environment. Pain associated with wounds and procedure discomfort significantly decreased, and analgesics were either reduced or eliminated in all patients. In a prospective case series by Holubova et al., nine outpatient patients indicated that MGH treatment positively influences the healing process of infected nonhealing lesions of various etiologies. The case report by Yang et al. describes the successful treatment of a patient with a palatal ulcer following segmental Le Fort I osteotomy, utilizing medical-grade honey, a liquid diet, local topical honey ointment, and hyperbaric oxygen therapy (HBO). Twenty-five researchers want to improve the composition and processing of this *Trigona* honey to attain Medical Grade Honey designation. This aims to improve and maximize the antibacterial properties of the patient's oral wound healing process.

## 4. Conclusion

Honey serves as a natural alternative to conventional wound dressings. The composition of different chemicals can signify various signs in wound healing. Honey may expedite the healing process following palatoplasty. Enhancing the biochemical activity of *Trigona* honey is essential to optimize its efficacy in wound healing, particularly for oral cavity injuries.

## Figures and Tables

**Table 1:** Literature on comparison of honey with other ingredients in palate wounds and/or palatoplasty management.

Studies	Population	Type of intervention in study	Comparison	Outcome
Zhafirah et al. [9]	45 Sprague Dawley rats	Punch biopsy on the hard palate with Premier Uni-Punch width 3 mm The treatment of each group 25 mg was given every morning until the fourth day. Tissue surrounding the biopsy incision was taken as 15 mg	Black forest honey Aloclair Aquadest	The VEGF expression levels were as follows: honey group = 41.10 ± 0.26 ng/ml, Aloclair group = 39.57 ± 0.27 ng/ml, and aquadest group = 33.26 ± 0.62 ng/ml (*p* ≤ 0.01)

Adekunle et al. [10]	50 patients	Palatoplasty using two flap methods. As part of the postoperative care, a collective honey is administered 24 hours after the surgical procedure, for a duration of 2–4 weeks, with a dosage of 1 ml every 6 hours. Following every meal, the control group utilized a 20 ml warm saline solution for oral hygiene. A fresh warm saline solution, consisting of 20 ml, was prepared and initiated 24 hours after palatoplasty	Forever TM bee honey, produced by forever living products, is a type of natural honey Warm saline solution	After two weeks following the surgery, complete healing of the bilateral mucoperiosteal defect resulting from cleft palate repair was observed in 16 (66.7%) participants who received honey and in 10 (38.5%) participants who received saline. Nevertheless, the disparity did not demonstrate statistical significance (*χ*^2^ (1, 50) = 3.978, *p*=0.055)

Aida et al. [11]	30 Sprague Dawley rats	Punch biopsy on the hard palate to the exposed periosteum with Premier Uni-Punch width 3 mm. In each group, up to 30 mg of forest honey, Aloclair, and aquadest were applied topically to the wound area over three days. On the fourth day, tissue samples were collected to assess TGF-*β*1 protein expression levels	Forest honey Aloclair Aquadest	The honey treatment group showed a 2.19-fold increase in TGF-*β*1 expression compared to aquadest-treated group (NC) and a 1.07-fold increase in the Aloclair gel–treated group (PC) (*p* < 0.01). The Aloclair gel–treated group (PC) had higher TGF-*β*1 expression than aquadest-treated group (NC), with a difference of 2.04 (*p* value < 0.01)

Golpasandhagh et al. [12]	60 rats	Oral wounds created with 4 × 6 mm in size on the anterior palatal rugae. This study utilized Lotus Honey, derived from the nectar of the *Ziziphus*. After 24 hours, the oral wounds were cleansed with sterile saline. All treatments were repeated three times a day. During the treatment process, the rats were given a topical application of 0.1 ml per group. The rats were denied access to food and water for up to 2 hours following treatment.	Saline Honey gel (20% of polyethylene glycol and 80% of honey) Polyethylene glycol base Oral/edible honey	1. The number of neutrophil cells was high in all groups on day three and steadily declined on days 7 through 14. The control group (*p*=0.001), the honey gel group (*p*=0.04), and the oral honey group (*p*=0.04) all had significantly lower neutrophil counts on the seventh day than on the third day 2. Macrophage cells exhibited the highest abundance on day three and showed a gradual decrease from Day 3 to Day 7. This decline was statistically significant in the oral honey group, with a p value of 0.02. The decrease in the number of macrophage cells remained consistent from Days 7 to 14. This decrease was statistically significant in the control group, with a *p* value of 0.02 3. The blood vessel count in all groups exhibited a significant rise from Day 3 to Day 7. Specifically, the honey gel group showed a significant increase (*p*=0.01), as did the baseline group (*p*=0.02). On the 14th day, there was a notable and meaningful difference observed among the groups, with a statistically significant *p* value of 0.01 4. The quantity of fibroblast cells increased progressively on the third, seventh, and fourteenth days, and it was significant in all groups. There were notable increases observed between Days 7 and 14 in the control group (*p* < 0.004), oral honey group (*p* < 0.03), and basal group 5. There was a statistically significant increase in epithelialization for all groups between Day 3 and Day 7. Epithelialization considerably increased from Day 7 to Day 14 in the control (*p* < 0.02), honey gel (*p* < 0.01), and oral honey (*p* < 0.04) groups 6. Collagen fibers exhibited a progressive rise from the third to the seventh day in all groups. However, statistically significant increases were observed only in the honey gel group (*p* < 0.04) and the oral honey group (*p* < 0.01). During the period from the seventh to the fourteenth days, there was a significant rise in collagen fibers in all groups

Onah et al. [13]	115 patients	After regaining consciousness from anesthesia on the day of surgery, the patient was initially given clear fluids (sugared water) for feeding. This was followed by a semisolid diet consisting of pap for a duration of three weeks. Honey received positive reinforcement starting on the second day. The units varied in their guidelines on the initiation of feeding and the duration of a liquid diet. One unit allowed patients to consume clear liquids orally within 24 hours after the operation and regularly recommended consuming edible honey as a post-surgical measure. While some units allowed patients to consume clear liquids orally after 48 hours, they did not recommend the consumption of edible honey	Solid feed ≤ 1 month Solid feed > 1 month Consumption of edible honey No consumption of edible honey	Consequently, the incidence of wound breakdown is not exclusively attributed to random chance but is also associated with whether a patient consumed honey or not (*p* < 0.001)

Alasqah.et al. [14]	20 patients	The width and length of the donor location were measured and documented. The test group's donor site received Medihoney dressing material, while no dressing substance was used in the control group Postoperative instructions included a soft diet, one tablet of ibuprofen 600 mg every eight hours, and a twice-daily chlorohexidine 0.12% mouthwash	Medihoney dressing No dressing	1. During the initial week, there was a notable disparity in the percentage of patients who exhibited healing at the donor site. In the test group, 56% of patients had healing in both width and length, while in the control group, only 44% experienced healing in both width and length (*p*=0.001). After four weeks, the test group had a donor site healing rate of 86% in width and 91% in length, while the control group had a healing rate of only 14% in width and 9% in length. This difference was statistically significant, with a *p* value of 0.001 2. The test group experienced much less pain and discomfort (*p*=0.001) compared to the control group

## Data Availability

The data that support the findings of this study are openly available in Dental Journal at https://e-journal.unair.ac.id/MKG/article/view/36061, reference number doi:10.20473/j.djmkg.v56.i1.p48-52.

## References

[1] Toma A. I., Fuller J. M., Willett N. J., Goudy S. L. (2021). Oral Wound Healing Models and Emerging Regenerative Therapies. *Translational Research*.

[2] Naidu P., Yao C. A., Chong D. K., Magee W. P. (2022). Cleft Palate Repair: A History of Techniques and Variations. *Plastic and Reconstructive Surgery—Global Open*.

[3] Bangun K., Halim J., Tania V. (2022). Lima Protocol for Cleft Palate Repair in Cleft and Craniofacial Centre Cipto Mangunkusumo Hospital Indonesia: A Preliminary Study. *Jurnal Plastik Rekonstruksi*.

[4] Seswandhana R., Makrufardi F., Sudjatmiko G. (2021). Fistula Incidence after Primary Repair and Correlation With Cleft Width-To-Palatum Width Ratio: A Prospective Cohort Study. *Annals of Medicine and Surgery*.

[5] Iftikhar A., Nausheen R., Mukhtar I (2022). The Regenerative Potential of Honey: A Comprehensive Literature Review. *Journal of Apicultural Research*.

[6] Kanimozhi S., Aswini A., Yogapriya P., Geetha K. Novel Nanofibrous Honey as a Wound Dressing Material—A Review.

[7] Hunter M., Kellett J., D’Cunha N. M., Toohey K., McKune A., Naumovski N. (2020). The Effect of Honey as a Treatment for Oral Ulcerative Lesions: A Systematic Review. *Exploratory Research and Hypothesis in Medicine*.

[8] Yupanqui Mieles J., Vyas C., Aslan E., Humphreys G., Diver C., Bartolo P. (2022). Honey: An Advanced Antimicrobial and Wound Healing Biomaterial for Tissue Engineering Applications. *Pharmaceutics*.

[9] Halim J., Dwimartutie N. (2020). Honey Accelerates Wound Healing in Pressure Ulcer: A Review. *Jurnal Plastik Rekonstruksi*.

[10] Zhafirah R., Nur Aida A., Hirawan H., Wardana T. (2023). Wound Healing Induces VEGF Expression Stimulated by Forest Honey in Palatoplasty Sprague Dawley. *Dental Journal*.

[11] Adekunle A. A., James O., Onuoha E. O., Adeyemo W. L. (2022). Wound Healing Following Palatoplasty Using Either Honey or Warm Saline Mouth Bath for Postoperative Wound Care: A Randomized-Controlled Study. *The Cleft Palate-Craniofacial Journal*.

[12] Wardana T., Aida A., Zhafirah R., Hirawan H., Haris Budi Widodo A., Prihastuti C. (2022). Wound Healing Potential of Forest Honey for Increasing TGF-Β1 Protein Expression in Palatoplasty: In-Vivo and In-Silico Studies. *Scientific Dental Journal*.

[13] Golpasandhagh L., Salehi P., Karimi B., Moghimipour E. (2021). Therapeutic Effect of Ziziphus lotus Honey on Hard Palate Ulcers in Rats. *Medical Studies*.

[14] Vyas T., Gupta P., Kumar S., Gupta R., Gupta T., Singh H. (2020). Cleft of Lip and Palate: A Review. *Journal of Family Medicine and Primary Care*.

[15] Ruslin M., Dom L., Tajrin A (2019). Establishing Cleft Services in Developing Countries: Complications of Cleft Lip and Palate Surgery in Rural Areas of Indonesia. *Archives of Plastic Surgery*.

[16] Kreshanti P. (2018). Maxillary Growth Evaluation of Patients With Unilateral Complete Cleft Lip and Palate After Two Flap Palatoplasty With Honey Oral Drops. https://www.jprjournal.com.

[17] Lauren M. P., Marcus V. M., Da S., Sady C. (2010). Comparative Study of Three Techniques of Palatoplasty inPatients With Cleft of Lip and Palate via Instrumental and Auditory Perceptive Evaluations.

[18] Chhabra S., Chhabra N., Kaur A., Gupta N. (2017). Wound Healing Concepts in Clinical Practice of OMFS. *Journal of Maxillofacial and Oral Surgery*.

[19] Ismardianita E., Rosalina W. (2020). Acceleration of Granulation Tissue Using Myrmecodiapendens Extract Induction During Wound Healing Tooth Extraction Process (Experimental Research on Caviacobaya). *Journal of Dentomaxillofacial Science*.

[20] Von Den Hoff J. W., Maltha J. C., Kuijpers-Jagtman A. M. (2013). Palatal Wound Healing: The Effects of Scarring on Growth. *Cleft Lip and Palate: Diagnosis and Management*.

[21] Khan I., Cho N., Ahmed M., Ahmed O., Beg M. S. A. (2021). The Application of Buccal Fat Pad to Cover Lateral Palatal Defect Causes Early Mucolization. *Cureus*.

[22] Khairani R., Alamsyah Harahap R., Endah Ndolisa Ginting K., Amir A., Saputri K., Hutagaol J. (2023). Development of Trigona SP (Stingless Bee) Honey Bee Cultivation as an Alternative Economy in Urban Community (Urban Bee) at Kelurahan Kampung Baru Kecamatan Medan Maimun. *GANDRUNG: Jurnal Pengabdian Kepada Masyarakat*.

[23] Zulkifli N. A., Hassan Z., Mustafa M. Z (2023). The Potential Neuroprotective Effects of Stingless Bee Honey. *Frontiers in Aging Neuroscience*.

[24] Kreshanti P., Sudjatmiko G., Bangun K. (1970). The Effect of Honey Give as Oral Drops in Precipitating Epithelialization of Lateral Palatal Defects Post TwoFlap Palatoplasty. *Jurnal Plastik Rekonstruksi*.

